# Construction of a tumor immune infiltration macrophage signature for predicting prognosis and immunotherapy response in liver cancer

**DOI:** 10.3389/fmolb.2022.983840

**Published:** 2022-09-02

**Authors:** Anmin Huang, Bei Lv, Yunjie Zhang, Junhui Yang, Jie Li, Chengjun Li, Zhijie Yu, Jinglin Xia

**Affiliations:** ^1^ Key Laboratory of Diagnosis and Treatment of Severe Hepato-Pancreatic Diseases of Zhejiang Province, The First Affiliated Hospital of Wenzhou Medical University, Wenzhou, China; ^2^ Translational Medicine Laboratory, The First Affiliated Hospital of Wenzhou Medical University, Wenzhou, China; ^3^ The First Clinical Medical College, Wenzhou Medical University, Wenzhou, China; ^4^ Department of Radiation Oncology, Zhongshan Hospital, Fudan University, Shanghai, China; ^5^ Wenzhou Key Laboratory of Hematology, The First Affiliated Hospital of Wenzhou Medical University, Wenzhou, China; ^6^ Department of Intervention, The First Affiliated Hospital of Wenzhou Medical University, Wenzhou, China; ^7^ Liver Cancer Institute, Zhongshan Hospital, Fudan University, Shanghai, China

**Keywords:** macrophage, tumor microenvironment, immune infiltration, prognosis, immunotherapy response

## Abstract

Liver cancer is an extraordinarily heterogeneous malignant disease. The tumor microenvironment (TME) and tumor-associated macrophages (TAMs) are the major drivers of liver cancer initiation and progression. It is critical to have a better understanding of the complicated interactions between liver cancer and the immune system for the development of cancer immunotherapy. Based on the gene expression profiles of tumor immune infiltration cells (TIICs), upregulated genes in TAMs and downregulated genes in other types of immune cells were identified as macrophage-specific genes (MSG). In this study, we combined MSG, immune subtypes, and clinical information on liver cancer to develop a tumor immune infiltration macrophage signature (TIMSig). A four-gene signature (S100A9, SLC22A15, TRIM54, and PPARGC1A) was identified as the TAM-related prognostic genes for liver cancer, independent of multiple clinicopathological parameters. Survival analyses showed that patients with low TIMSig had a superior survival rate than those with high TIMSig. Additionally, clinical immunotherapy response and TIMSig was observed as highly relevant. In addition, TIMSig could predict the response to chemotherapy. Collectively, the TIMSig could be a potential tool for risk-stratification, clinical decision making, treatment planning, and oncology immunotherapeutic drug development.

## Introduction

The development is regulated by the dynamic tumor microenvironment (TME) which is comprised of a complex network of multipotent stromal cells, fibroblasts, blood vessels, endothelial cell precursors, immune cells, and various secreted factors such as cytokines (Anderson and Simon, 2020). Tumor immune infiltration cells (TIICs) have been demonstrated to be important in tumor proliferation, invasion, metastasis, and drug resistance through complex immune interaction with malignant cells (Binnewies et al., 2018; Lei et al., 2020). Recent studies have shown that TIICs are directly linked to the prognosis of cancer patients (Fridman et al., 2017; Giraldo et al., 2019). The temporal and spatial heterogeneity of TME in hepatocellular carcinoma (HCC) suggests that the disorder and imbalance of TME caused by various factors, such as DNA methylation and chromatin architecture, may be one of the most critical mechanisms of tumorigenesis and progression (Kurebayashi et al., 2018; Fu et al., 2019; Johnstone et al., 2020). At present, most studies on the occurrence and development of primary liver carcinoma focus on HCC, while little attention has been paid to cholangiocarcinoma (CCA) and combined hepatocellular-cholangiocarcinoma (cHCC-CCA). Herein, we integrate the information from all pathological types of liver cancer to establish a novel risk assessment model to evaluate the risk of liver cancer.

Macrophages that reside within the TME are known as tumor-associated macrophages (TAMs). As the predominant infiltrated TIICs, TAMs promote tumor development at multiple levels, such as accelerating genomic mutation, cultivating tumor stem cells, paving the way for metastasis, and taming the immune system (Wynn et al., 2013; Mantovani et al., 2017). There are two types of macrophages classically activated pro-inflammatory (M1) and alternatively activated anti-inflammatory (M2) phenotypes. TAMs can be polarized into M2 phenotype macrophages or M1 phenotype macrophages by different stimuli (Vitale et al., 2019; Yoon et al., 2019). M1 macrophages release various pro-inflammatory cytokines, active oxygen, and nitrogen oxide that drive tumor-killing activities (Qian and Pollard, 2010). In contrast, factors such as IL-10, TGF-β, and VEGF secreted by M2 macrophages promote tumor progression, angiogenesis, metastasis, and suppression of anti-tumor immunity (Lujambio et al., 2013). Previous studies on TAMs have suggested that high M2 macrophage infiltration is associated with a poor prognosis in cancers (Martínez et al., 2017; Väyrynen et al., 2021). Therefore, TAMs are correlated to the development of liver tumors and the clinical prognosis of patients, and a TAM-based signature could have potential application in predicting clinical outcomes and immunotherapeutic responses.

Immune checkpoint inhibitors (ICIs) have made an indelible mark in the field of cancer immunotherapy (Vaddepally et al., 2020). ICI immunotherapy covers a series of monoclonal antibodies designed to block the binding of immune checkpoints expressed on the surface of immune cells to their ligands, which eliminates the immunosuppression caused by immune checkpoints and revitalizes the function of T cells (Dyck and Mills, 2017). ICIs such as anti-cytotoxic Tlymphocyte associated protein 4 (CTLA4, CTLA-4), anti-programmed cell death protein 1 (PD1, PD-1), and anti-programmed cell death one ligand 1 (PDL1, PD-L1) have been approved for the clinical application of advanced malignancies, including melanoma, non-small-cell lung cancer, urothelial carcinoma, gastric cancer, liver cancer, and Hodgkin’s lymphoma (Wei et al., 2018; Perez-Ruiz et al., 2019). Although ICIs have demonstrated their efficiency and durability in the treatment of solid tumors, a great number of patients have limited benefits in terms of response and survival (Syn et al., 2017; Schoenfeld and Hellmann, 2020; Bagchi et al., 2021). At present, some research perspectives are that this phenomenon may be related to T cell depletion and mechanical factors of TME, which place restrictions on the efficacy of ICI treatment in cancer patients (Sacks et al., 2018; Wong et al., 2021). Therefore, new molecules and prognostic models should be explored to predict or improve the clinical response and application of ICI therapy.

**FIGURE 1:**
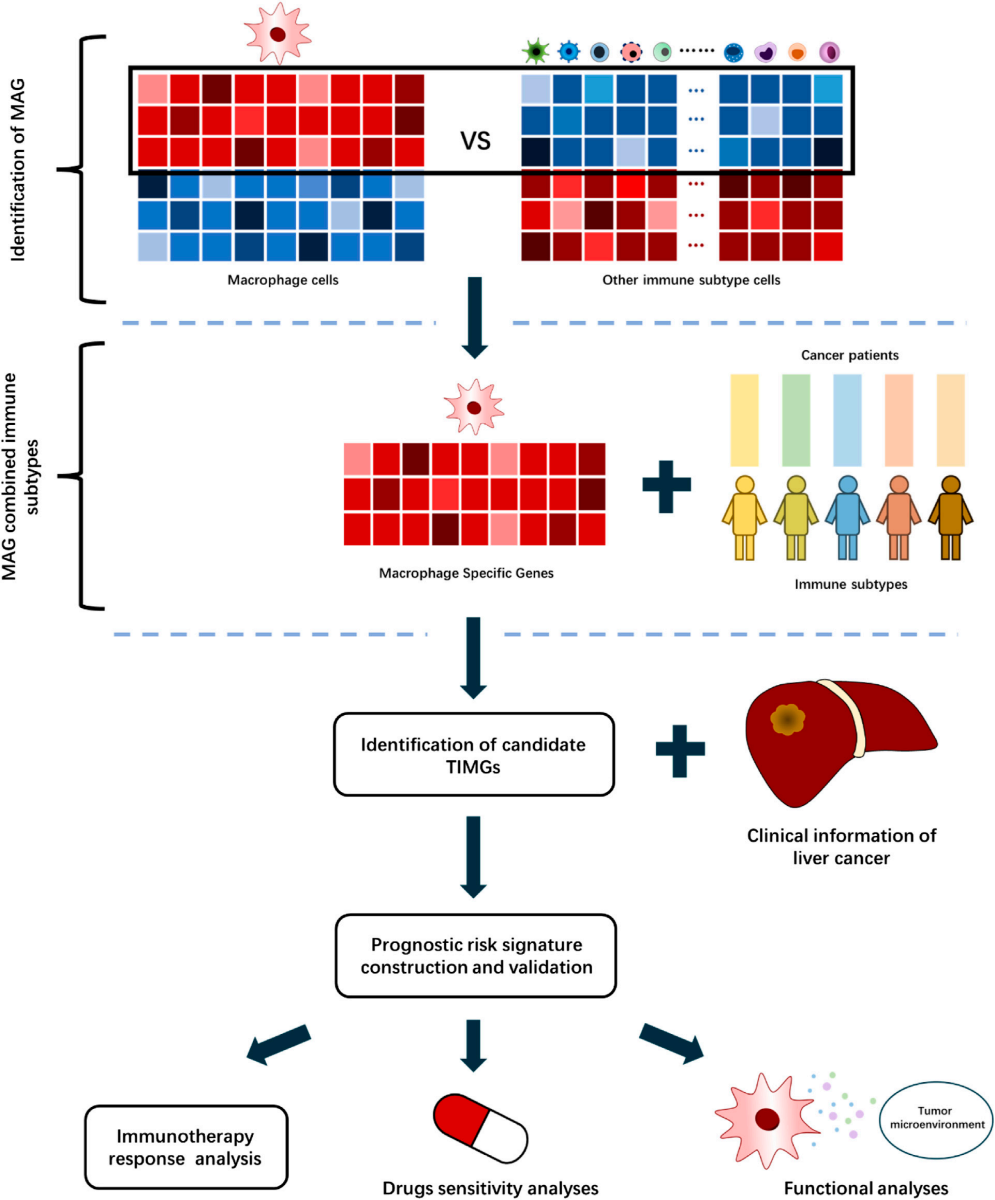
Workflow of TIMSig generation and validation.

This study developed a computational algorithm framework for identifying prognostic signatures with TAMs (Figure 1). We selected different immune datasets from the Gene Expression Omnibus (GEO, https://www.ncbi.nlm.nih.gov/geo/database) database, which contains bulk RNA-sequencing data of cell lines, primary animal cells, and primary human cells, for screening the tumor immune infiltration macrophage genes (TIMGs). We performed a systematic and thorough biomarker analysis and validation to locate and construct a risk predictive signature for the prognosis of liver cancer by taking into account multiple components of TME. Herein, we report a tumor immune infiltration macrophage signature (TIMSig), which can predict the prognosis and immunotherapy of liver cancer individuals and reflect the cellular functions of macrophages.

## Methods

### Data collection

The transcriptome sequencing information and corresponding clinical data (*n* = 404) of liver cancer samples were obtained from The Cancer Genome Atlas (TCGA, https://portal.gdc.cancer.gov/). The gene expression data were given in log2 (x+1) transformed and multiple imputations with R package “mice” prior to analyses. Transcriptional profiles of macrophages from three datasets (GSE158792, GSE56755, and GSE75829) and 21 other immune cell profiles from 15 datasets (GSE23371, GSE27291, GSE157737, GSE27838, GSE37750, GSE8059, GSE51540, GSE149425, GSE104852, GSE52156, GSE155148, GSE83441, GSE42058, GSE106932, and GSE28726) were obtained from the GEO database, these profiles were used to perform differential gene analyses using GEO2R online tools with R package “limma”. The intersection of upregulated genes in macrophages (cut-off was set at logFC >1.5, *p* < 0.05) and downregulated genes (cut-off was set at logFC < -1.5, *p* < 0.05) in other immune cells was considered as macrophage-specific genes (MSG).

### Identification and prognostic assessment of immune subtypes in liver cancer

CIBERSORT algorithm was performed to characterize 22 immune cell subtypes of immune landscape using the deconvolution strategy. The 404 liver cancer patients from TCGA were clustered according to immune landscape by using the R package “ConsensusClusterPlus”, and a consensus matrix was constructed to define the immune subtypes. The prognostic potential of each immune subtype was estimated by Kaplan–Meier (K-M) curves. Then, the association between the MSG and different immune subtypes of cancer patients was investigated by using the R package “limma”, with |logFC| > 1.5 and *p* < 0.05 considered as significantly different.

### Construction and validation of the TIMSig

Cancer cases from the TCGA database were randomized into the training cohort or test cohort for the construction and validation of risk scores at the ratio of 1:1 (202 in the training set, 202 in the test set). In the training cohort, we used univariate Cox proportional hazards regression analysis and LASSO regression analysis to investigate the prognostic performance of candidate TIMGs. Then, TIMGs were determined by multivariate Cox proportional hazards regression analysis, and the risk score based on TIMGs was constructed. The samples were divided into the high-risk group and low-risk group based on the median risk score. The difference in overall survival (OS) between the high-risk group and low-risk group was performed using the log-rank test. Various statistical methods, including K–M curves, univariate Cox proportional hazards regression analysis, multivariate Cox proportional hazards regression analysis, and time-dependent receiver-operating characteristic (ROC) curves were performed to identify the association between TIMSig and OS.

### Validation of the TIMSig in the GSE14520 cohort and ICI clinical samples

Another sample information of the GSE14520 dataset included information from 242 tumor samples, which was downloaded from the GEO database. Due to different platforms, only part of four TIMGs in the TILSig was covered by the other dataset. Therefore, the risk score is only based on part of the TIMGs. Moreover, we collected 24 PD-1/PD-L1 clinical treated cases with HCC from GSE140901 with available progression-free survival (PFS) records based on the nCounter PanCancer Immune Profiling Panel platform, which came from the National Taiwan University Hospital for estimation of the potential of TIMGs as immunotherapy biomarkers. The association between TIMSig and PD-1/PD-L1 immunotherapy response was investigated by paired t-test. ROC curve was performed to estimate the accuracy of TIMSig response to predict ICI treatment response. K–M curve was performed to evaluate the risk-stratification capability of TIMSig and PD-1/PD-L1 immunotherapy response.

### Tumor immune microenvironment analyses

ESTIMATE algorithm was performed to calculate the scores of immune infiltration and tumor purity based on gene expression profile. The ESTIMATE analysis method is integrated into the “estimate” R package. Tumor Immune Estimation Resource 2.0 (TIMER, http://timer.comp-genomics.org/) is an excellent tool to assess the level of immune infiltration, applied to analyze the association between TIMGs and abundance of TIICs for estimation of the capability of TIMGs in predicting TIICs. In addition, the expression differences of TIMGs between cancer and normal samples were visualized.

### Functional analyses

We carried out the R package “ggpubr” to investigate the function of macrophages in secreting cytokines by analyzing the association of the TIMSig and the expression level of corresponding cytokine molecules. Then, The Gene Set Enrichment Analysis (GSEA, ver. 4.1.0) was applied to analyze the organism function and cell development with TIMSig through the Gene Ontology (GO) and the Kyoto Encyclopedia of Genes and Genomes (KEGG) databases. The Human Protein Atlas (HPA, https://www.proteinatlas.org/) provides immunohistochemistry (IHC) information for human cancers, which applied the HPA database to evaluate the protein expression levels for TIMGs.

### Statistical analyses

All statistical analyses were performed by R version 4.1.1, in addition to corresponding R packages and online tools. A *p*-value < 0.05 was considered statistically significant. Univariate Cox proportional hazards regression analysis and multivariate Cox proportional hazards regression analysis methods were integrated in the R package “survival” and “survminer”. LASSO Cox proportional regression analysis was performed with the R package “glmnet”. ROC curves were performed, and the area under curves (AUC) was applied to estimate the prognostic performance of TIMSig using the R package “survivalROC”. The relationship between the TIMSig and immune checkpoints was evaluated to verify the potential prognostic capacity of TIMSig for ICI immunotherapy. Drug sensitivity analysis was applied with the R package “pRRophetic”.

## Results

### Identification of MSG

**FIGURE 2:**
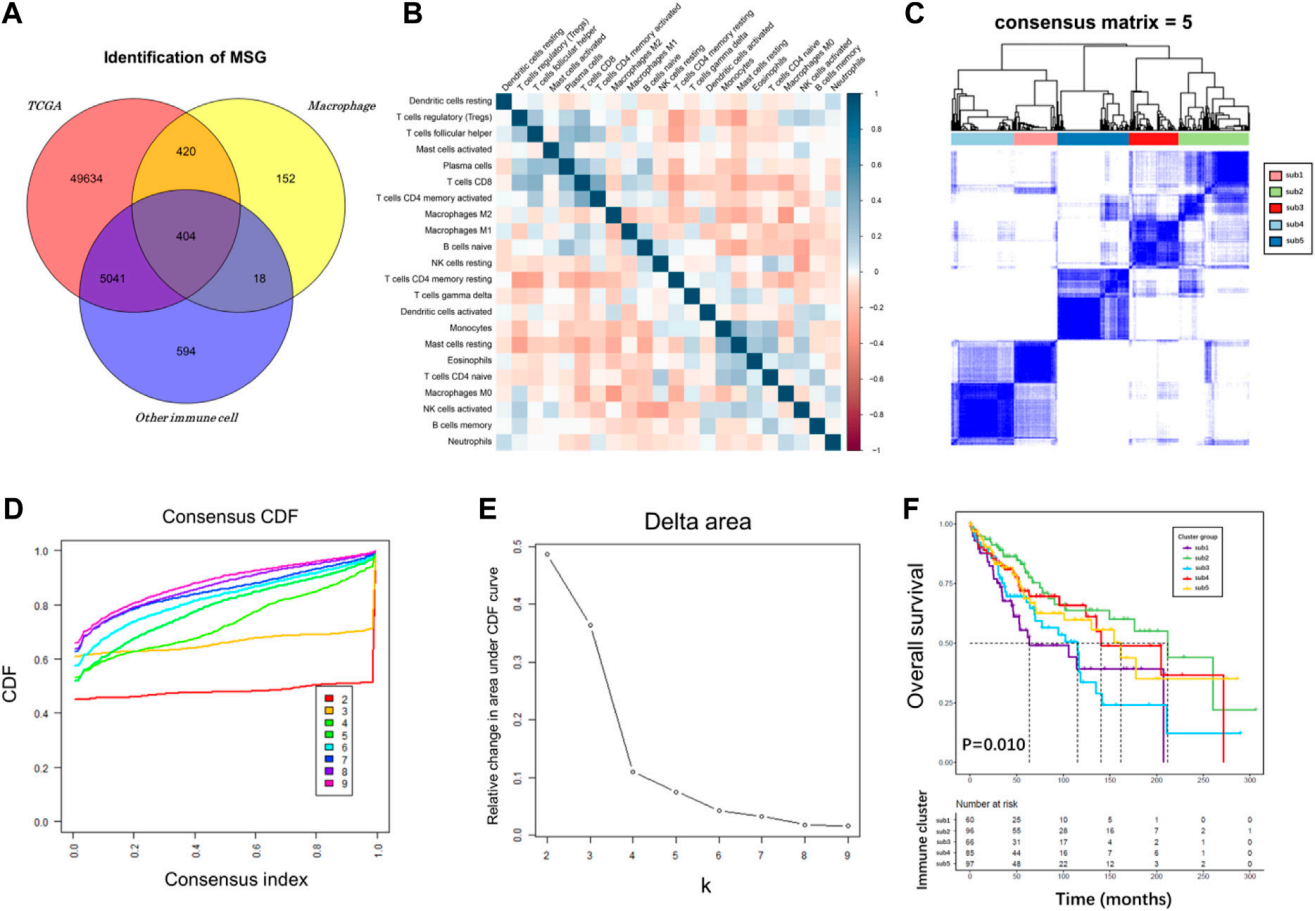
Immune subtype analysis of TCGA cohort. **(A)** Venn diagram plot among TCGA, macrophage, and other immune cell genes. **(B)** Correlation heatmap for TCGA cancer samples based on the CIBERSORT immune infiltration algorithm. **(C–E)** Consensus score matrix for cancer samples when k = 5. A higher consensus score between two samples indicates that they are more likely to be assigned to the same cluster in different iterations. **(F)** A K-M survival curve of the five immune subtypes.

To characterize the gene expression pattern in various immune cell subtypes, we retrieved 18 expression profiles of immune cells from the GEO database. Differential expression analyses were performed on each dataset with “limma” algorithm, including macrophages (GSE158792, GSE56755, and GSE75829) and other immune cells (GSE23371, GSE27291, GSE157737, GSE27838, GSE37750, GSE8059, GSE51540, GSE149425, GSE104852, GSE52156, GSE155148, GSE83441, GSE42058, GSE106932, and GSE28726). The platform information of these datasets was shown in Supplementary Table S1. Subsequently, 994 upregulated macrophage genes and 6,057 downregulated genes were considered as statistically significantly different in TIICs. Then, 422 dysregulated genes were identified that were both highly upregulated in macrophages and downregulated in other immune cells, demonstrating their expression specificity for TAMs rather than other types of immune cells. Finally, 404 MSG were screened and combined with clinical features for the construction of a prognostic risk model (Figure 2A).

### Identification and prognostic assessment of immune subtypes in liver cancer

Immunotyping can be used to mirror the immune status in TME and help identify suitable genes for cancer immunotherapy. The immune landscape based on the CIBERSORT algorithm was applied to investigate the immune subtypes. We compared median survival rates among these subtypes in the TCGA cohorts by setting *p* < 0.05 as the threshold for screening. The correlation heatmap of the CIBERSORT immune infiltration profile was shown in Figure 2B. We chose k = 5 where immune infiltration appeared to be stably clustered (Figures 2C–E), and obtained five immune subtypes that had a significant difference for OS (Figure 2F, *p* = 0.010). These results support a distinct immune infiltration feature of five immune subtypes, reflecting clinical outcomes due to immune infiltration disorder and its predictive value in classifying liver cancer patients. The association of MSG and immune subtypes was performed with R package “limma”. The cut-off was set at |logFC| >1.5, *p* < 0.05, adjust *p* < 0.05.

### Identification and verification of the prognostic capability of the TIMSig

**FIGURE 3:**
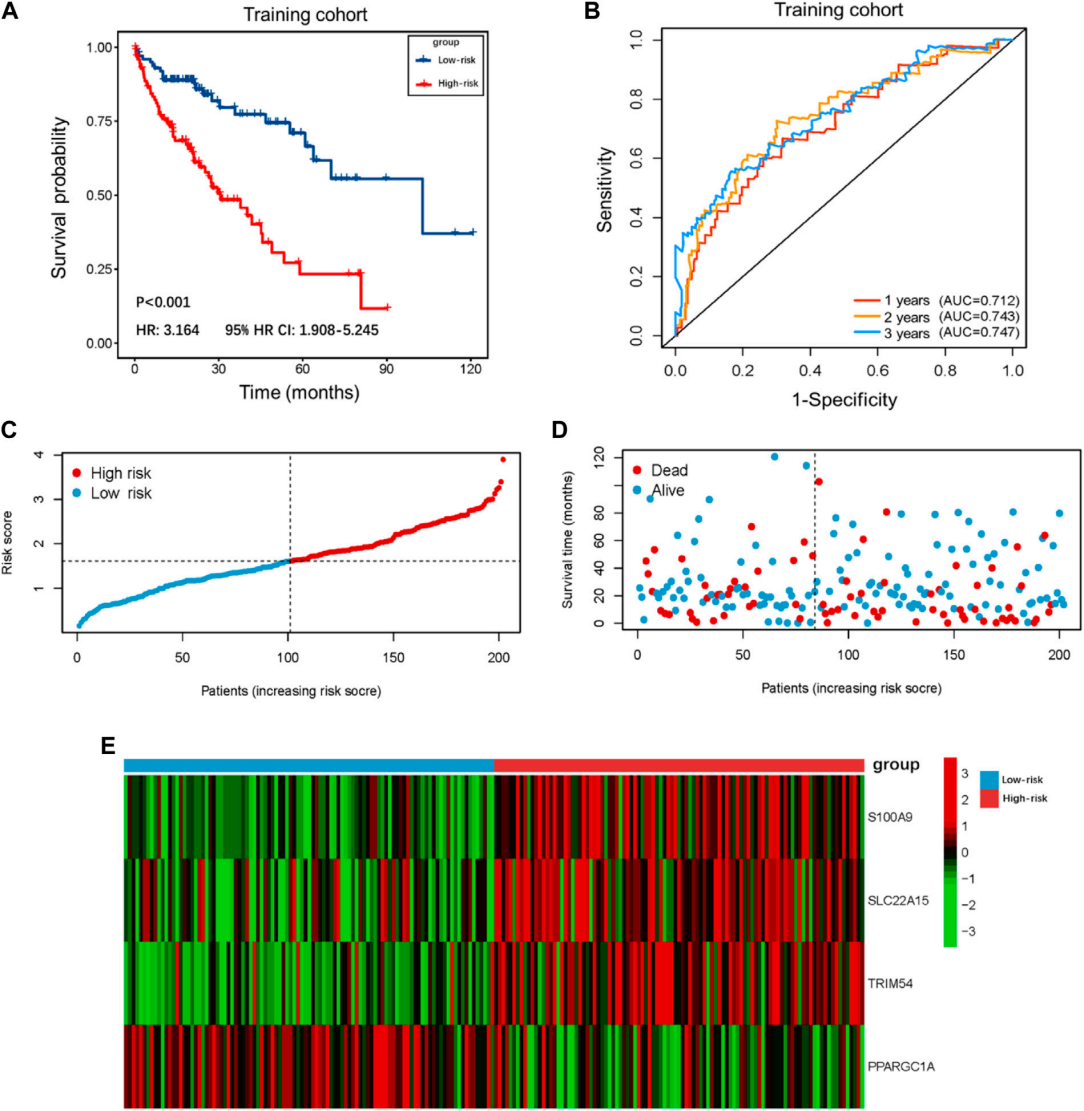
Survival analysis of the training cohort. **(A)** K–M curve for the training cohort. Patients in the low-risk group represented a better OS. **(B)** The 1, 2, and 3 years ROC curves of the risk score in the training cohort. **(C)** Distribution of risk score of liver cancer patients in the training cohort. **(D)** Distribution of survival status of liver cancer patients in the training cohort. **(E)** Heatmap of the TIMGs in the training cohort.

**FIGURE 4:**
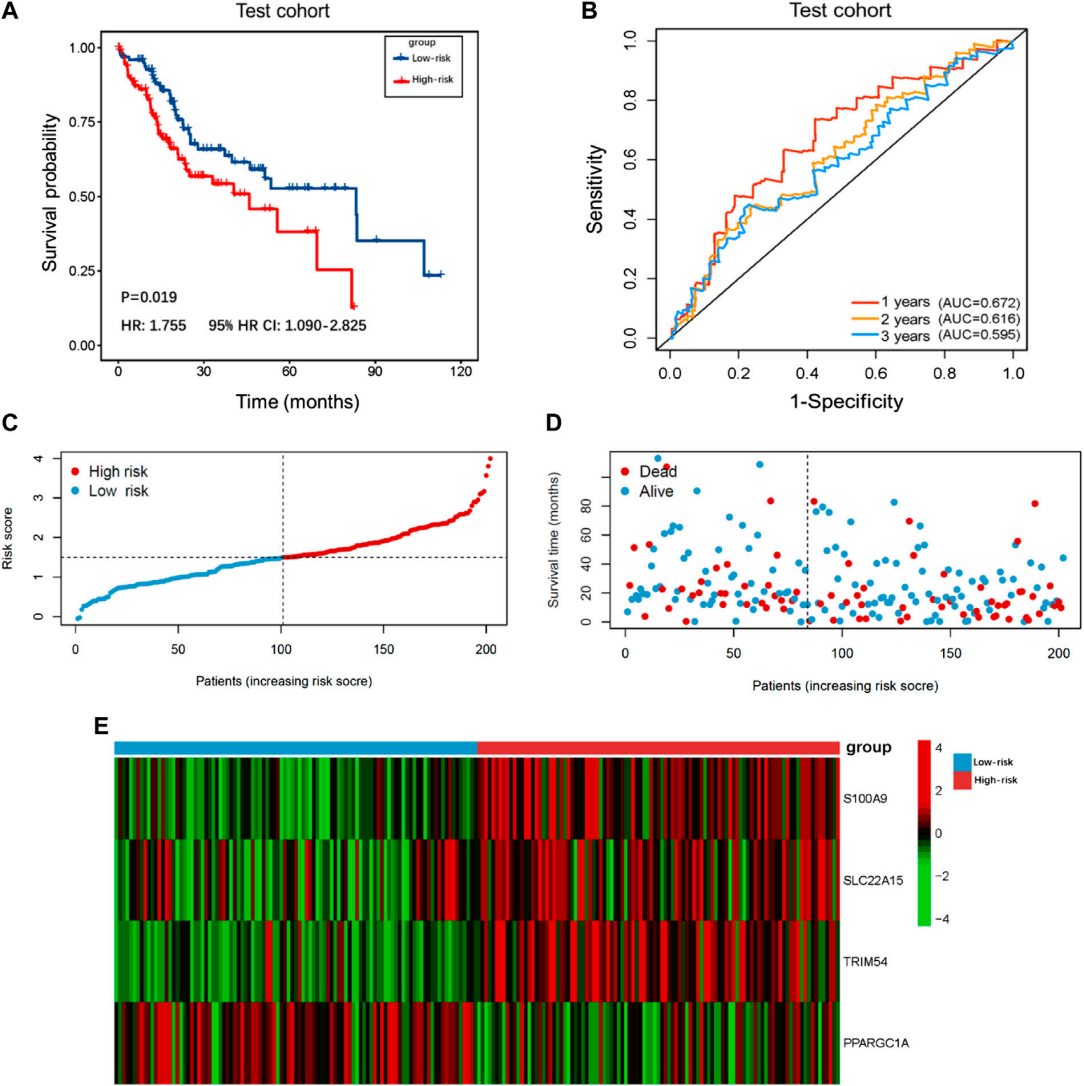
Survival analysis of the test cohort. **(A)** K–M curve for the test cohort. Patients in the low-risk group represented a better OS. **(B)** The 1, 2, and 3 years ROC curves of the risk score in the test cohort. **(C)** Distribution of risk scores of liver cancer patients in the test cohort. **(D)** Distribution of survival status of liver cancer patients in the test cohort. **(E)** Heatmap of the TIMGs in the test cohort.

**FIGURE 5:**
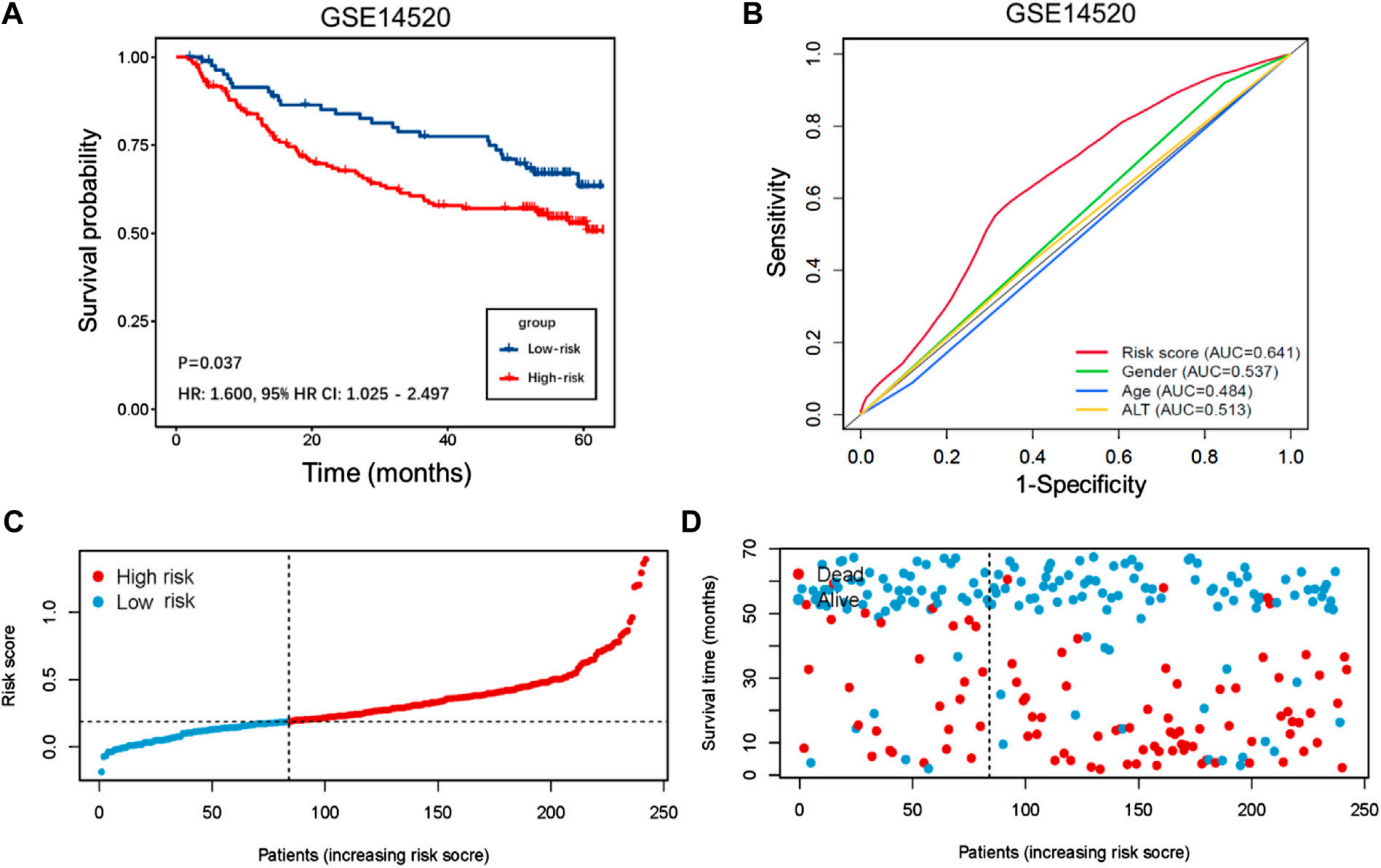
Validation of GSE14520 cohort. **(A)** K–M curve for the GSE14520 cohort. Patients in the low-risk group represented a better OS. **(B)** Comparison of the 3 years' ROC curve with other clinical characteristics. **(C)** Distribution of risk score of liver cancer patients in the GSE14520 cohort. **(D)** Distribution of survival status of liver cancer patients in the GSE14520 cohort.

The demographic and clinical characteristics of patients in the two cohorts were similar (Supplementary Table S2). In the training cohort (*n* = 202), we used univariate Cox proportional hazards regression analysis, LASSO regression analysis (Supplementary Figures S1A,B), and multivariate Cox proportional hazards regression analysis to develop TIMSig. The following formula was used for calculation: risk score = (0.170 × S100A9) + (0.090 × SLC22A15) + (0.143 × TRIM54) + (-0.088 × PPARGIA). Cancer patients were classified into different groups according to respective median risk scores. With the increase in the risk scores, the number of liver cancer patients in the high-risk group increased (Figures 3C,D, Figures 4C,D, and Figures 5C,D). In the training cohort (*n* = 202), the K–M survival curve demonstrated that patients in the low-risk group had a significantly longer OS compared to those in the high-risk group (Figure 3A, *p* < 0.001, HR: 3.164, 95% HR CI: 1.908–5.245). The AUC of ROC curves for 1, 2, and 3 years was higher than 0.7 in the training cohort (Figure 3B). Similar trends were found in the test cohort (*n* = 202), the high-risk group exhibited a shorter survival time in liver cancer patients compared to those in the low-risk group (Figure 4A, *p* = 0.019, HR: 1.755, 95% HR CI: 1.090–2.825). According to the results of ROC curves, the AUC for 1, 2, and 3 years was 0.672, 0.616, and 0.595 in the test cohort, respectively (Figure 4B). To further verify the prognosis prediction capability of TIMSig, the prognostic performance of the TIMSig was further tested using another completely independent GEO cohort with the GSE14520 dataset (*n* = 242). As shown in the Figure 5A, the K–M curve for OS showed a statistically significant difference between the two groups (*p* = 0.037, HR: 1.600, 95% HR CI: 1.025–2.497). Patients tend to have a better prognosis in the low-risk group compared to the high-risk group. In Figure 5B, the AUC of the ROC curve was 0.641 for 3 years, which was significantly higher than other clinical features.

### The TIMSig is an independent prognostic factor in liver cancer patients

**FIGURE 6:**
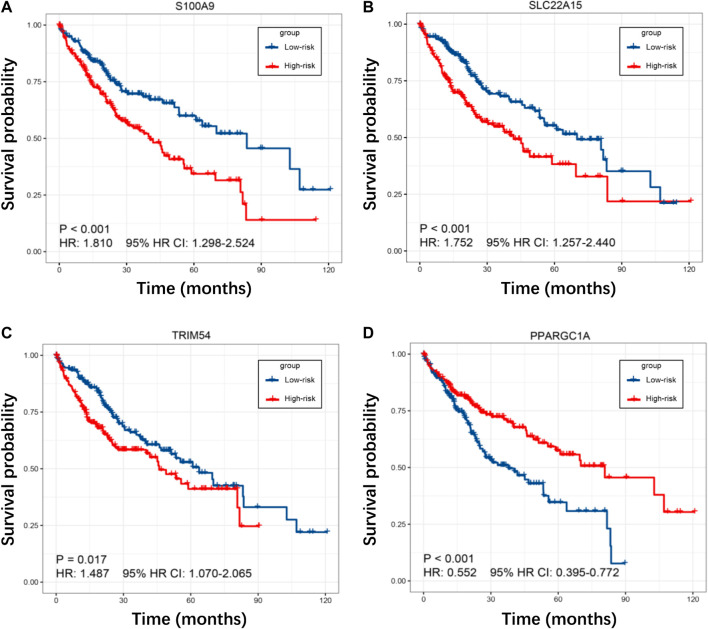
**(A–D)** K–M curves for S100A9, SLC22A15, TRIM54, and PPARGC1A in the TCGA cohort.

To investigate the prognostic power of our risk model, the TIMSig was performed as an independent prognostic factor in univariable and multivariable Cox analyses for each cohort (Table 1). For the training cohort, univariate Cox regression analysis showed that risk score (*p* < 0.001, HR: 2.717, 95% HR CI: 1.963–3.760) and stage (*p* = 0.003, HR: 1.443, 95% HR CI: 1.133–1.837) were significantly related to the prognosis of liver cancer. Multivariate Cox regression analysis confirmed that the risk score (*p* < 0.001, HR: 2.562, 95% HR CI: 1.854–3.540) still was an independent prognostic factor after adjusting for other clinicopathologic factors. Then, the role of TIMSig was validated by using the test cohort, the results of univariate Cox regression analysis (*p* = 0.006, HR: 1.529, 95% HR CI: 1.132–2.067) and multivariate Cox regression analysis (*p* = 0.014, HR: 1.493, 95% HR CI: 1.084–2.057) for OS represented that risk score were significantly relevant to the clinical outcomes of liver cancer. For the GSE14520 cohort, the TIMSig still were an independent prognostic predictor for OS in the univariate Cox regression analysis (*p* = 0.022, HR: 2.179, 95% HR CI: 1.118–4.249) and multivariate Cox regression analysis (*p* = 0.019, HR: 2.282, 95% HR CI: 1.146–4.545). Taken together, the TIMSig can be applied to predict the prognosis of patients with liver cancer and has a great accuracy, which is consistent across different cohorts. Moreover, patients with a low expression value of each TIMGs had longer survival times than patients in the high-risk group (Figures 6A–D).

**TABLE 1:** Univariate Cox analysis and multivariate Cox analysis of OS in each cohort.

	Univariate analysis			Multivariate analysis		
Variables	HR	95% HR CI	*p* value	HR	95% HR CI	*p* value
Training cohort						
Risk score	2.717	1.963–3.760	<0.001	2.562	1.854–3.540	<0.001
Stage	1.443	1.133–1.837	0.003	1.305	1.018–1.673	0.036
Gender (female/male)	1.079	0.662–1.758	0.760	1.141	0.694–1.878	0.603
Age (>65/≤65)	1.381	0.869–2.193	0.172	1.252	0.780–2.009	0.352
BMI (>30/≤30)	0.618	0.332–1.151	0.129	0.557	0.297–1.044	0.068
Test cohort						
Risk score	1.529	1.132–2.067	0.006	1.493	1.084–2.057	0.014
Stage	1.558	1.211–2.004	0.001	1.449	1.114–1.885	0.006
Gender (female/male)	0.644	0.406–1.021	0.061	0.730	0.448–1.189	0.206
Age (>65/≤65)	1.219	0.766–1.939	0.403	1.073	0.664–1.735	0.773
BMI (>30/≤30)	1.365	0.791–2.357	0.264	1.408	0.805–2.463	0.230
GSE14520 cohort						
Risk score	2.179	1.118–4.249	0.022	2.282	1.146–4.545	0.019
Gender (female/male)	1.859	0.901–3.834	0.093	1.930	0.932–3.996	0.077
Age (>65/≤65)	0.726	0.352–1.497	0.386	0.793	0.383–1.641	0.532
ALT (>50/≤50 U/L)	1.155	0.772–1.727	0.483	1.113	0.743–1.666	0.604

### Potential of the TIMSig as an indicator of immunotherapy response in liver cancer

**FIGURE 7:**
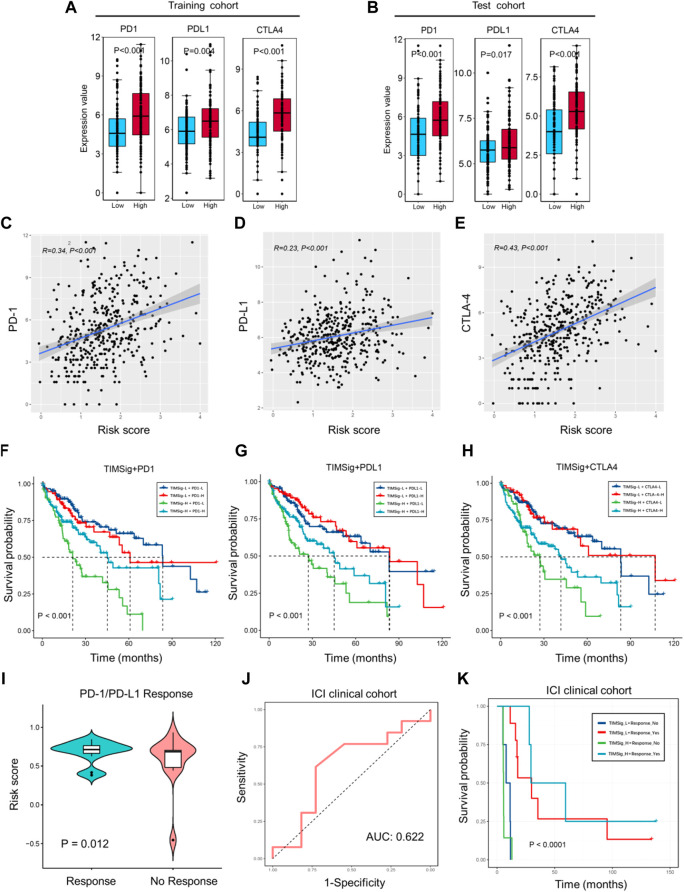
Immunotherapy response with TIMSig. **(A,B)** Comparison of the expression value of immune checkpoints (PD1, PDL1, and CTLA4) with different groups of TIMSig in each cohort. **(C–E)** The Pearson correlation between immune checkpoints and the risk scores in the TCGA cohort. **(F–H)** K-M survival curves of OS among four patient groups stratified by the TIMSig and PD1, PDL1, and CTLA4 in the TCGA cohort. **(I)** Comparison of the risk score with different groups of PD1/PDL1 therapy responses, paired t-test was used as the significance test. **(J)** The ROC curve to estimate the sensitivity of TIMSig to PD1/PDL1 therapy responses. **(K)** K–M survival curves of PFS among four patient groups stratified by the TIMSig and PD1/PDL1 therapy reponses.

Given the success of anti-PD1, anti-PDL1, and anti-CTLA4 treatments in cancer patients, we compared the difference in the expression value of immune checkpoints (including PD-1, PD-L1, and CTLA-4), and the different subgroups categorized by the median of TIMSig. In the training cohort, patients with high TIMSig tend to show higher expression value of immune checkpoints compared with low TIMSig (Figure 7A, *p* < 0.001, *p* = 0.004, *p* < 0.001 for comparison of PD-1, PD-L1, and CTLA-4 with each group). Also, the patients with the high TIMSig still show higher expression value of immune checkpoints compared with low TIMSig in the test cohort, (Figure 7B, *p* < 0.001, *p* = 0.017, *p* < 0.001 for comparison of PD-1, PD-L1, CTLA-4 with each group).

To verify whether the TIMSig has an impact on OS in liver cancer patients with the homologous trend of immune checkpoints and different risk groups. Based on the TIMSig and the expression value of immune checkpoints, the survival distribution of total TCGA patients was partitioned into four groups and compared by log-rank test. We found that TIMSig was positively related to PD-1 (Figure 7C, R = 0.34, *p* < 0.001), PD-L1 (Figure 7D, R = 0.23, *p* < 0.001), CTLA-4 (Figure 7E, R = 0.43, *p* < 0.001). As shown in Figure 7F, patients with a low level of PD-1 and high TIMSig would have significantly worse OS than the other three groups (*p* < 0.001), whereas patients with high TIMSig and a low level of PD-L1 were inclined to be the worst clinical outcomes relative to other three groups (Figure 7G, *p* < 0.001). The same statistical difference with OS was repeated using TIMSig and CTLA-4 (Figure 7H, *p* < 0.001). It is indicated that patients with liver cancer stratified by TIMSig and PD-L1 or CTLA-4 exhibited OS analogous to PD-1.

The 24 clinical cases of anti-PD1/PDL1 clinical treatment for HCC patients were obtained from GSE140901. A total of 13 PD-1/PD-L1 responses (54.2%) occurred, while 11 cases (45.8%) have nonresponses on PD-1/PD-L1 therapy. Based on the results of different analyses by paired t-test (Figure 7I, *p* = 0.012), TIMSig is significantly associated with PD-1/PD-L1 therapy response. The AUC value of the model established in the ICI clinical cohort is 0.622 and is a great predicted value for the estimation of clinical immunotherapeutic efficacy (Figure 7J). In addition, TIMSig combined PD-1/PD-L1 response can significantly stratify PFS (Figure 7K, *p* < 0.0001). These observed associations between the TIMSig and immune checkpoints confirmed our hypothesis that the TIMSig may be a great predictive biomarker for cancer immunotherapy response.

### The pertinence of TIMGs and immune infiltration level in liver cancer

**FIGURE 8:**
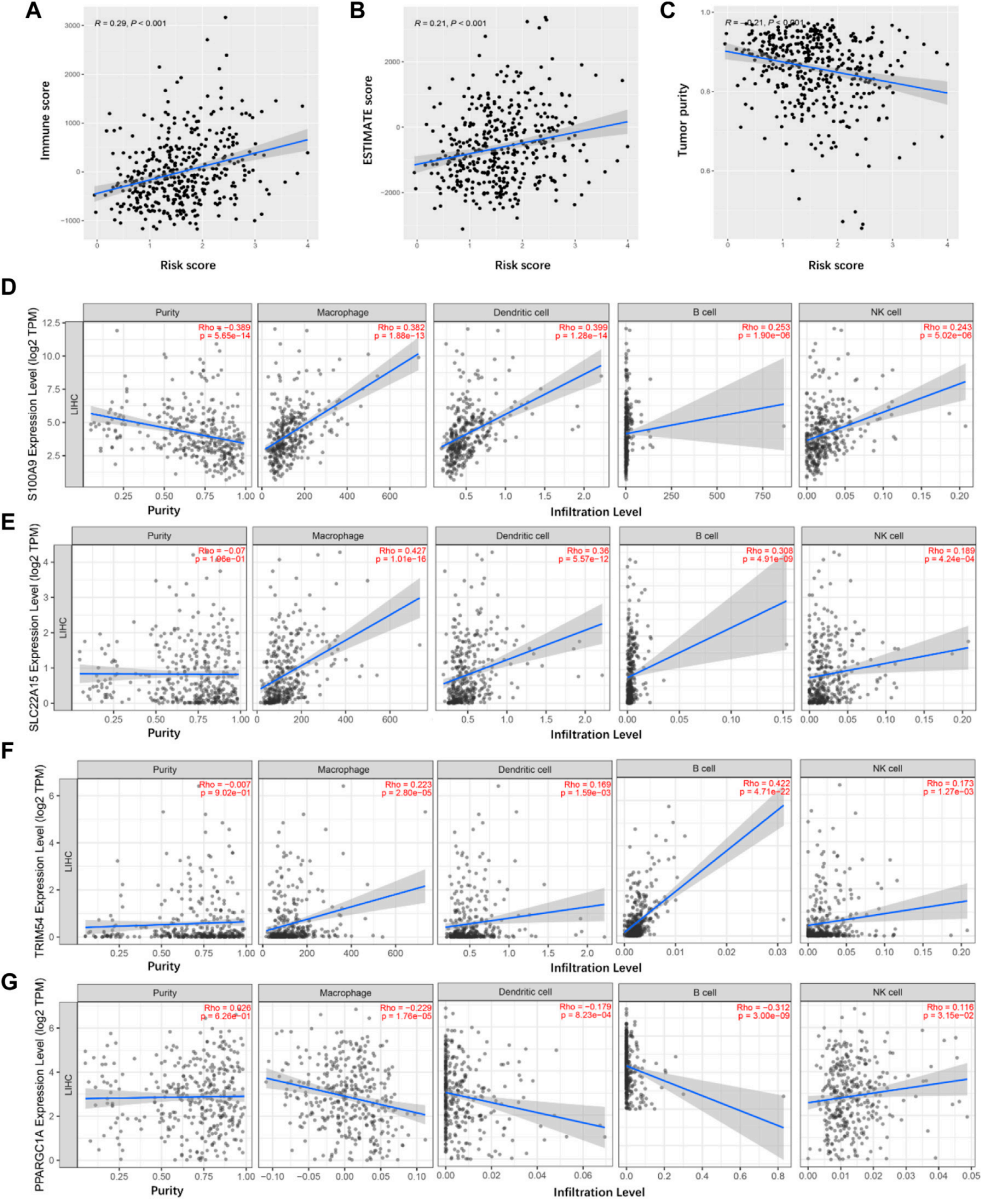
Tumor immune microenvironment analyses. **(A)** Correlation between the immune score and risk score in TCGA cancer samples. **(B)** Correlation between the ESTIMATE score and risk score in TCGA cancer samples. **(C)** Correlation between the tumor purity and risk score in TCGA cancer samples. **(D–G)** Identification of TIMGs associated with TIICs.

ESTIMATE algorithm gave scores for 404 TCGA cancer samples, containing the immune score, ESTIMATE score, and tumor purity. We compared the distribution of these scores with TIMSig to immune characteristics. Pearson correlation tests were applied while each specific score and TIMSig was inspected and analyzed as shown in Figures 8A–C. TIMSig was positively associated with immune score (R = 0.29, *p* < 0.001) and ESTIMATE score (R = 0.21, *p* < 0.001), while TIMSig and tumor purity was negatively correlated (R = -0.21, *p* < 0.001). To evaluate whether TIMGs can accurately predict the distribution of TIICs, the association of TIMGs expression level with TIICs’ abundance was evaluated using the TIMER2.0 database. It suggested that high expression levels of S100A9, SLC22A15, TRIM54, and PPARGC1A were significantly correlated with increased infiltration degree of macrophages, DCs, B cells, and NK cells (Figures 8D–G). Therefore, TIMGs may directly or indirectly present the immune cells in triggering an immune response.

### Functional analyses for TIMGs

**FIGURE 9:**
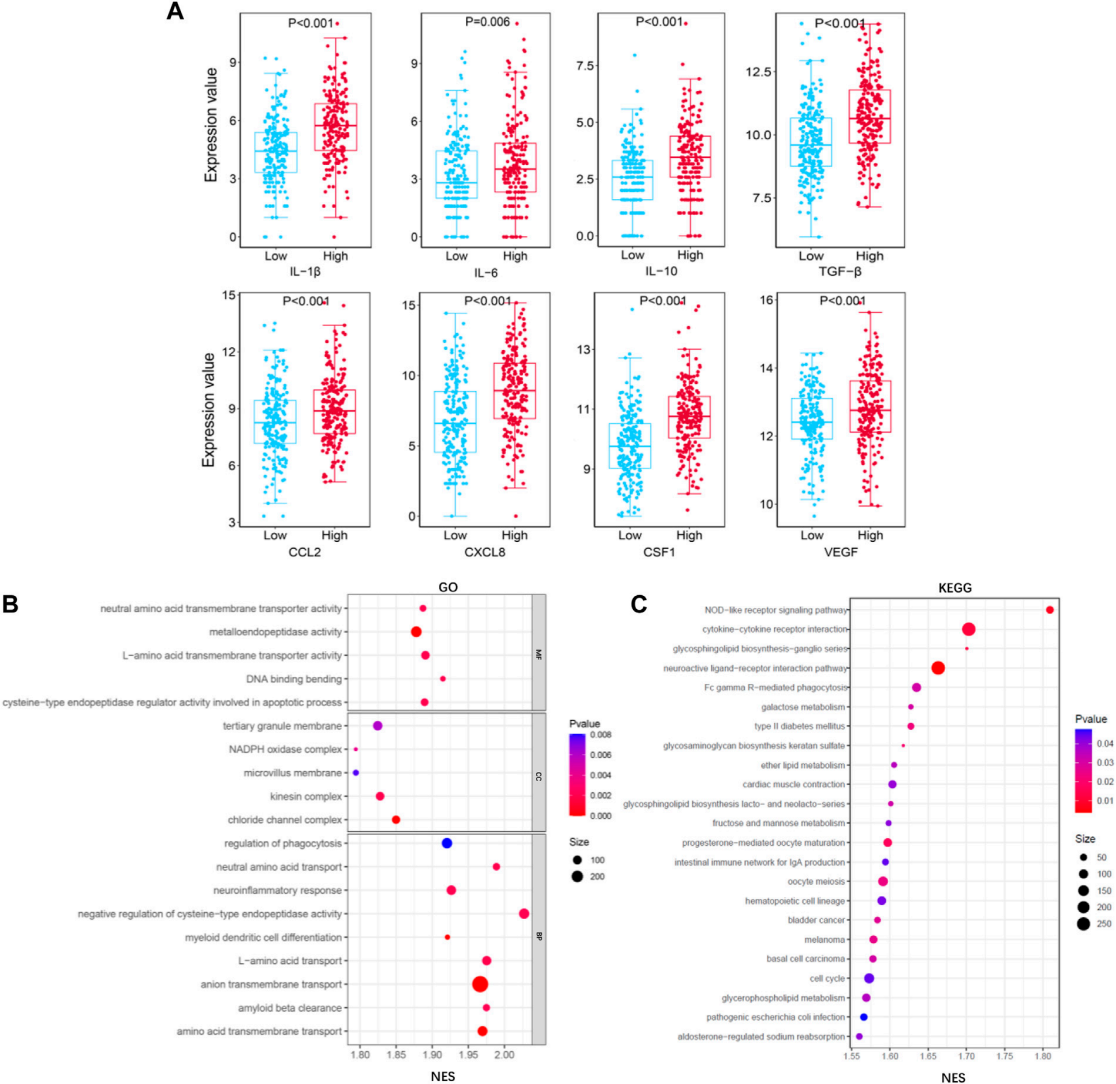
Functional analysis for TIMGs. **(A)** Comparison of the expression value of cytokines (IL-1β, IL-6, IL-10, TGF-β, CCL2, CXCL8, CSF-1, and VEGF) with different groups of TIMSig in the TCGA cohort. **(B)** GO function analysis of TIMSig in the TCGA cohort. **(C)** KEGG pathway analysis of TIMSig in the TCGA cohort.

The main role of TAMs is to accelerate tumor growth by secreting many cytokines. To analyze the function of macrophages in liver cancer tissues, we analyzed the relationship between TIMSig and cytokine gene expression. As shown in Figure 9A, eight cytokines (IL-1β, IL-6, IL-10, TGF-β, CCL2, CXCL8, CSF-1, and VEGF) were identified to have higher expression values in the high-risk group than in the low-risk group, which was relevant to the progression, invasion, and metastasis of cancers. The GO analysis of the biological process (BP), molecular function (MF), and cell component (CC) showed that most of the enriched terms were related to cellular material transport and material metabolism (Figure 9B). The KEGG pathway enrichment analysis illustrated that the TIMSig were mainly enriched in NOD-like receptor signaling pathway, cytokine–cytokine receptor interaction, and Fc gamma R-mediated phagocytosis, most of which are related to immunity and metabolism (Figure 9C). Concurrently, the protein level of S100A9, SLC22A15, and TRIM54 was found to be much higher in the cancer tissues or the cells around the blood sinus compared to the normal tissues (Supplementary Figure S2).

### Screening of sensitive chemotherapy drugs

**FIGURE 10:**
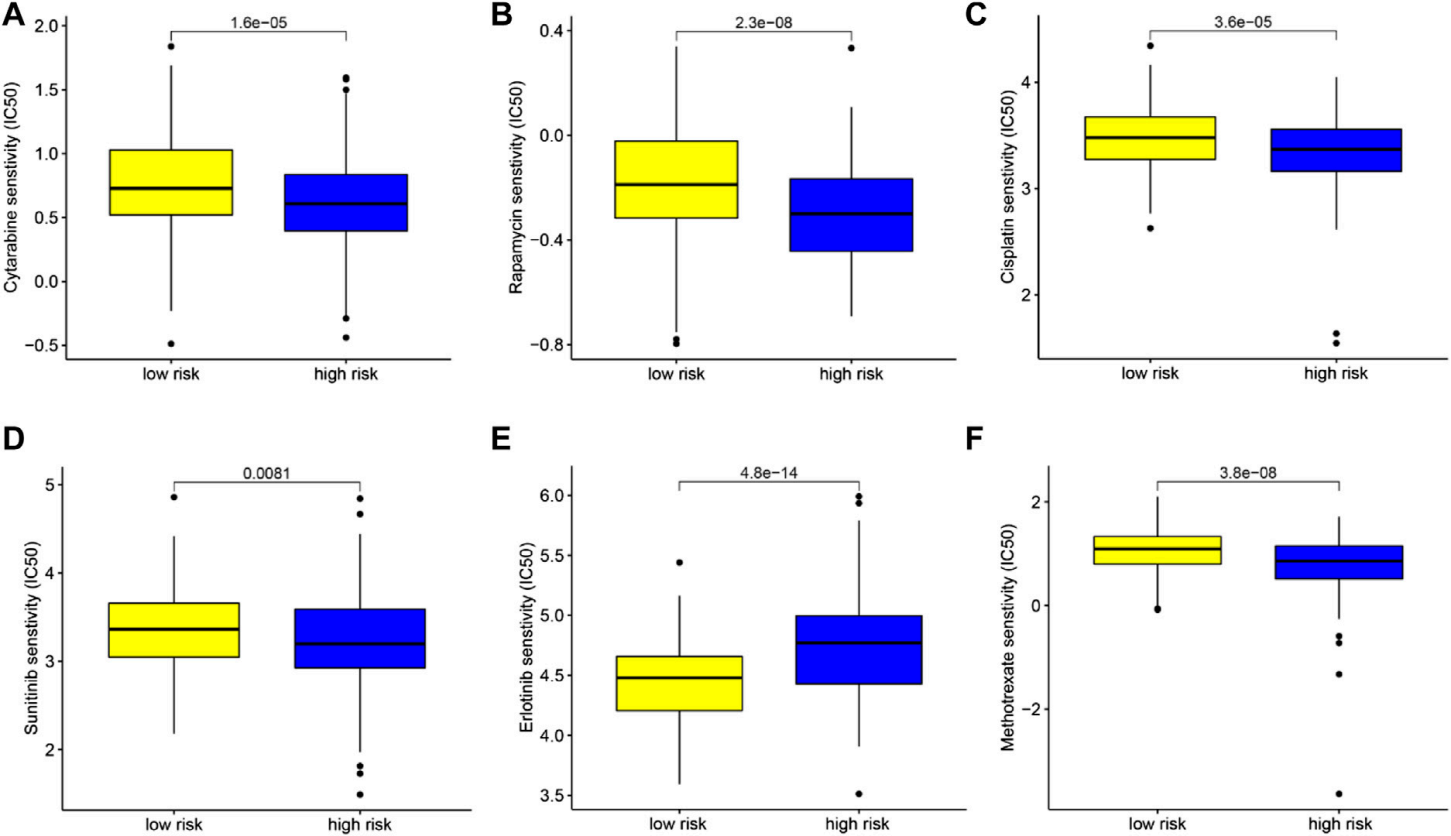
Chemotherapeutic drug sensitivity analysis based on TIMSig. **(A–F)** Comparison of the IC50 levels (cytarabine, rapamycin, cisplatin, sunitinib, erlotinib, and methotrexate) with different groups of TIMSig in the TCGA cohort.

Based on the pRRophetic algorithm, we explored the relationship between TIMSig and drug chemoresistance by calculating the half-maximal inhibitory concentration (IC50) of six common chemotherapeutic drugs (cytarabine, rapamycin, cisplatin, sunitinib, erlotinib, and methotrexate) for liver cancer. In Figures 10A–F, we observed that cancer patients in the high-risk group were more resistant to erlotinib. On the contrary, the patients with a high score of TIMSig were more sensitive to cytarabine, rapamycin, cisplatin, sunitinib, and methotrexate.

## Discussion

Liver cancer is the leading cause of cancer-related death in the world, due to its characteristics of late diagnosis, poor prognosis, and high heterogeneity (Heinrich et al., 2021; Sung et al., 2021). Hence, identifying reliable and effective biomarkers for liver cancer prognosis is of great importance. The tumor's immune status largely influences the effectiveness of cancer immunotherapy. Accumulating evidence has suggested that distinct molecular subtypes of tumors are positively associated with OS (Zhang et al., 2020; Hu et al., 2021; Liu et al., 2021). In this study, we defined five immune infiltration subtypes and screened TAM-related molecular biomarkers, which could help to predict the clinical response of ICI treatments.

TAMs can promote malignant cell proliferation by interacting with cancer cells by secreting ingredient exosomes and cytokines. Accumulating evidence has indicated that TAMs in TME is associated with the prognosis and immunological characteristics of a variety of cancers (Lam et al., 2019; Li et al., 2019; Xiao N et al., 2021). TAMs can mediate PD-1 drug resistance in HCC through the PD-L1 pathway and regulation of T cells (Pu and Ji, 2022). Molecular biomarkers of TAMs could be used for risk-stratification and response prediction of cancer treatments, though the fundamental cognition of cellular structures and molecular landscapes in liver cancer remains difficult to define. Traditional strategies for clinical models perform investigation mainly at the single component level and have inherent limitations in providing precise prognostic information on complex component cells residing in a highly multicomponent TME. In previous investigations, the signatures for prognostic prediction based on immune-related genes or immune microenvironment have been described in many kinds of cancers, and several mRNAs or lncRNA-associated signatures have also been developed to predict the clinical outcomes of HCC patients (Xiao B et al., 2021; Kang et al., 2021; Pi et al., 2021). However, the potential ability of TAMs for immunotherapy prediction has been ignored. In this study, we used the transcriptome profile of TAM to construct a risk model that could reflect the function of macrophages.

In our results, S100A9, SLC22A15, TRIM54, and PPARGC1A were identified as TIMGs, which were identified as prognostic biomarkers for liver cancer sufferers. The expression of these TIMGs in the training cohort and test cohort was shown in Figure 3E and Figure 4E. In addition, based on the pan-cancer analyses, TIMGs are differentially expressed not only in patients with liver cancer, but also in many other cancers (Supplementary Figure S3). In most cancer types, TIMGs exhibited an upregulated tendency in cancer samples compared to normal samples. To date, studies on S100A9 and PPARGC1A have been suggested as a new potential biomarker for liver cancer. S100A9, a secreted protein related to the inflammatory immune microenvironment and the functional phenotype of macrophages, is significantly increased in TAMs of HCC (Ganta et al., 2019). As a downstream regulator of VEGFR1 macrophage polarization, S100A9 is a promoter of M2 polarization. It has been found that after the knockout of S100A9, the levels and activities of CX3CR1 and Nur77 in macrophages decreased significantly, which resulted in the decrease of efferocytosis in macrophages and the accumulation of necrotic cells in tissues (MarinkoviÄ‡ et al., 2020; Willers et al., 2020). The existence of S100A9 positive macrophages in tumor tissues, a key gene in the growth and metastasis of HCC, was related to the shorter survival time and the poor treatment of PD-1 antibody in metastatic cancer patients (Duan et al., 2018; Kwak et al., 2020; Wei et al., 2021). PPARGC1A is a mitochondrial regulator, which can regulate mitochondrial biogenesis in macrophages and play a regulatory role in the growth and metastasis of liver cancer. The up-regulation of PPARGC1A will enhance the oxidation of fatty acids in mitochondria to reduce the accumulation of free fatty acids, which leads to abnormal mitochondrial function (Soliman et al., 2020). PPARGC1A mediates YAP to reprogram cell metabolism, shifting substrates from gluconeogenesis to growth anabolism (Hu et al., 2017). At the same time, it effectively inhibits aerobic glycolysis by adjusting Wnt/β-catenin/pdk1 axis, thus inhibiting the migration and invasion of HCC. Meanwhile, PPARGC1A interacts with TNFAIP3 and HSPA12A, produces nuclear translocation, induces AOAH expression, participates in mitochondrial regulation and the expression of inflammatory genes such as NLRP3, and plays a vital role in the homeostasis of telomeres and macrophages’ mitochondria (Kang et al., 2018; Liu et al., 2020).

TAM promotes intravasation, extravasation, and metastasis of tumor cells by secreting various pro-tumoral molecule proteins. Macrophages change their functional state by responding to signaling molecules in the TME, and secrete various cytokines (such as IL-1β, IL-6, CXCL-8, IL-10, CCL2, and CSF-1) to interact with various types of cells in the TME, thus playing a momentous role in the regulation of tumor invasiveness, metastasis and drug resistance (Galdiero et al., 2018; Lin et al., 2019; Korbecki et al., 2020). A common feature of the nine cytokines in Figure 9 is the enhancement of tumor progression *via* efferocytosis of TAMs which is essential for understanding the immune status of TME and improving the prognosis of liver cancer patients. It is proved that TIMGs are significantly associated with the biological functions of the immune system and will be a promising tool reflecting the function of TAMs in liver cancer. Thus, we consider that TIMSig plays a role in the crosstalk between the macrophages and the TME of liver cancer. For further studies, the connection among TIMGs, cytokines, and corresponding pathways could be an interesting direction.

Many previous studies have identified meaningful immune infiltration signatures for cancer patients (Chen et al., 2021; Lin et al., 2021; Xu et al., 2021). However, the immune subtype or the interaction between TAMs and immune cells in cancer metabolism was rarely considered. Compared with other similar prognostic models based on bioinformatics, our signature passed the test of clinical PD-1/PD-L1 treated cohort and has good relevance with PFS, which is the biggest advantage of our model. In addition, our study not only screened the immune subtypes of liver cancer based on immune infiltration, but also investigate the potential TIMSig-based regulatory mechanisms where the immune system participated and allowed robust risk-stratification, thus enhancing a broader notion of the TME-based prognostic model. Finally, based on the drug resistance algorithm, we explored six chemotherapeutic drugs that were related to TIMSig, which could provide guidelines for clinical cancer treatments. Except for macrophages, our algorithm is also suitable for identifying the specific genes of other immune cells in the prognosis of tumor patients.

There are several limitations to our study. The components of tumor tissues were complicated, it is not certain whether the collected cases and algorithm could accurately reflect the function of TAMs on the survival of liver cancer sufferers. In addition, our prognostic model is only based on bulk RNA-sequencing data and retrospective clinical cohort, and validation of this model by single-cell sequencing or flow cytometry might give a higher potential for its application. Finally, the mechanism of TRIM54 and SLC22A15 affecting liver cancer development through TAMs is still unclear, and further research was warranted.

## Conclusion

Overall, S100A9, SLC22A15, TRIM54, and PPARGC1A were screened as TIMGs that can be used for prognostic prediction and be the potential targets of the ICI treatments for patients with liver cancer. The prediction model integrated specific genes of immune cells in the TME will help clinicians not only make rational immunotherapeutic decisions but also understand the driving nodes in the machinery of liver cancer.

## Data Availability

Publicly available datasets were analyzed in this study. These data can be found at: GEO (https://www.ncbi.nlm.nih.gov/geo/database), TCGA (https://portal.gdc.cancer.gov/), CIBERSORT (https://cibersort.stanford.edu/), TIMER2.0 (http://timer.comp-genomics.org/), GSEA (http://www.gsea-msigdb.org/gsea/index.jsp), HPA (https://www.proteinatlas.org/).
