# Erratum: Construction of a tumor immune infiltration macrophage signature for predicting prognosis and immunotherapy response in liver cancer

**DOI:** 10.3389/fmolb.2022.1094902

**Published:** 2022-12-01

**Authors:** Frontiers Production Office

**Affiliations:** Frontiers Media SA, Lausanne, Switzerland

**Keywords:** macrophage, tumor microenvironment, immune infiltration, prognosis, immunotherapy response

Due to a production error, there was a mistake in Figures 6−10 as published. The image intended for Figure 10 was captioned as Figure 6, which resulted in Figures 6−9 to be transposed to captions 7–10. The corrected figures and captions appear below.

**FIGURE 6:**
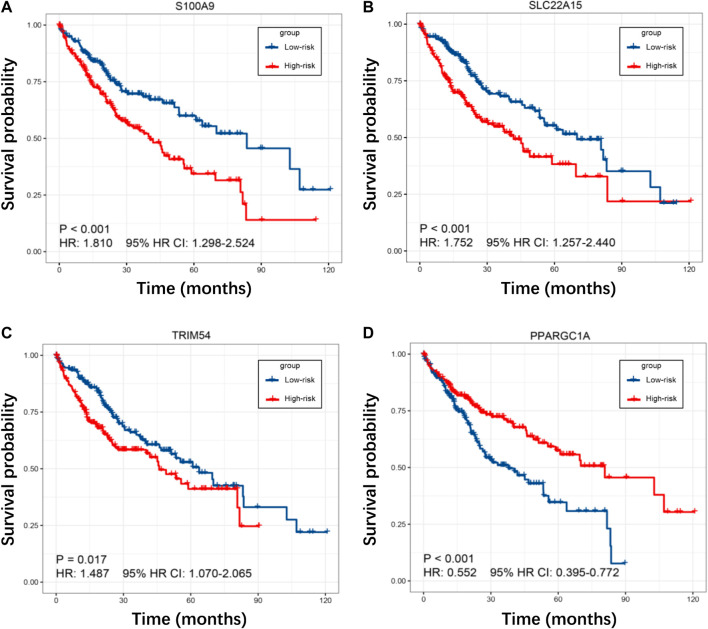
**(A–D)** K–M curves for S100A9, SLC22A15, TRIM54, and PPARGC1A in the TCGA cohort.

**FIGURE 7:**
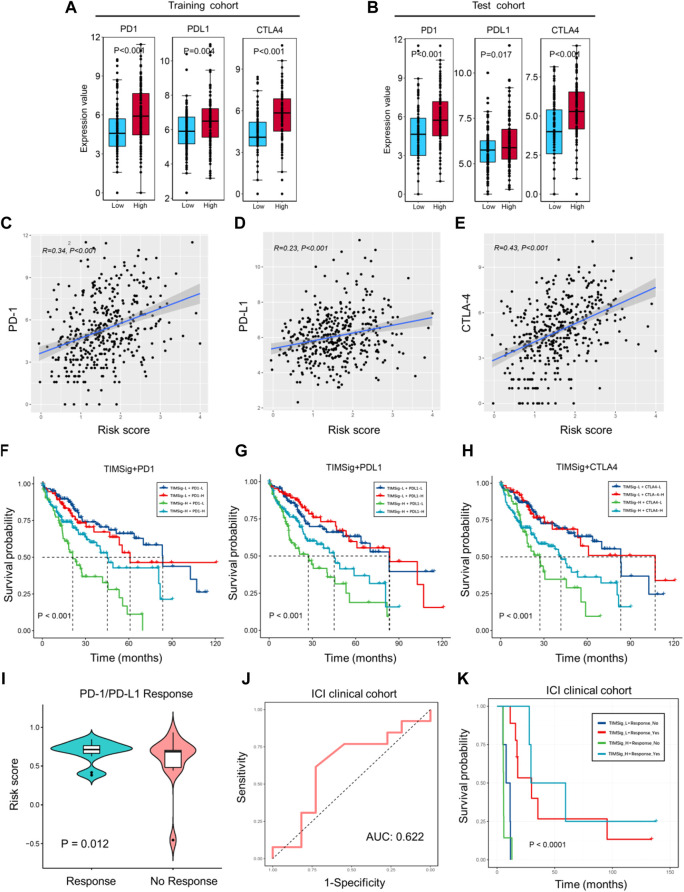
Immunotherapy response with TIMSig. **(A,B)** Comparison of the expression value of immune checkpoints (PD1, PDL1, and CTLA4) with different groups of TIMSig in each cohort. **(C–E)** The Pearson correlation between immune checkpoints and the risk scores in the TCGA cohort. **(F–H)** K-M survival curves of OS among four patient groups stratified by the TIMSig and PD1, PDL1, and CTLA4 in the TCGA cohort. **(I)** Comparison of the risk score with different groups of PD1/PDL1 therapy responses, paired t-test was used as the significance test. **(J)** The ROC curve to estimate the sensitivity of TIMSig to PD1/PDL1 therapy responses. **(K)** K–M survival curves of PFS among four patient groups stratified by the TIMSig and PD1/PDL1 therapy reponses.

**FIGURE 8:**
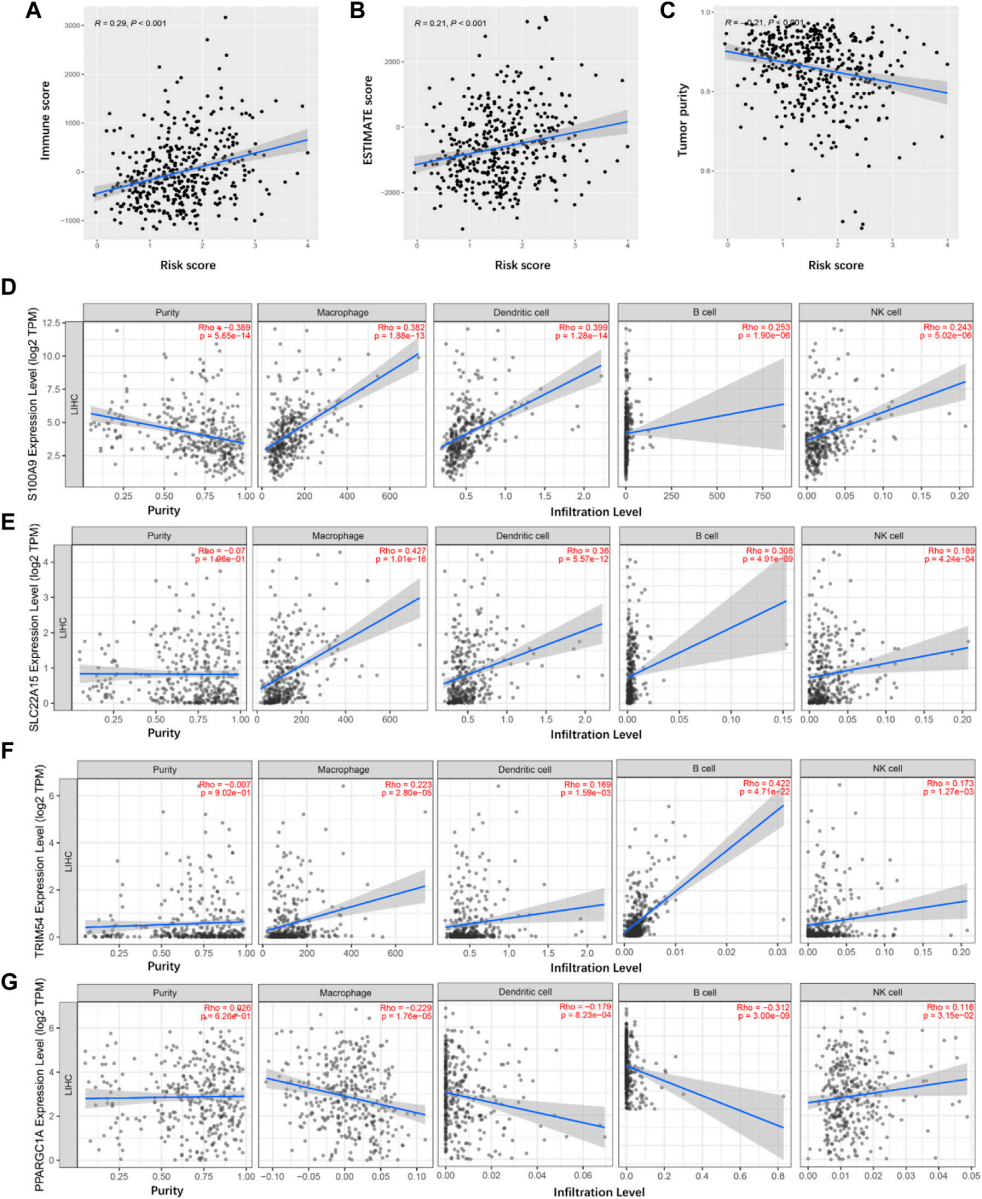
Tumor immune microenvironment analyses. **(A)** Correlation between the immune score and risk score in TCGA cancer samples. **(B)** Correlation between the ESTIMATE score and risk score in TCGA cancer samples. **(C)** Correlation between the tumor purity and risk score in TCGA cancer samples. **(D–G)** Identification of TIMGs associated with TIICs.

**FIGURE 9:**
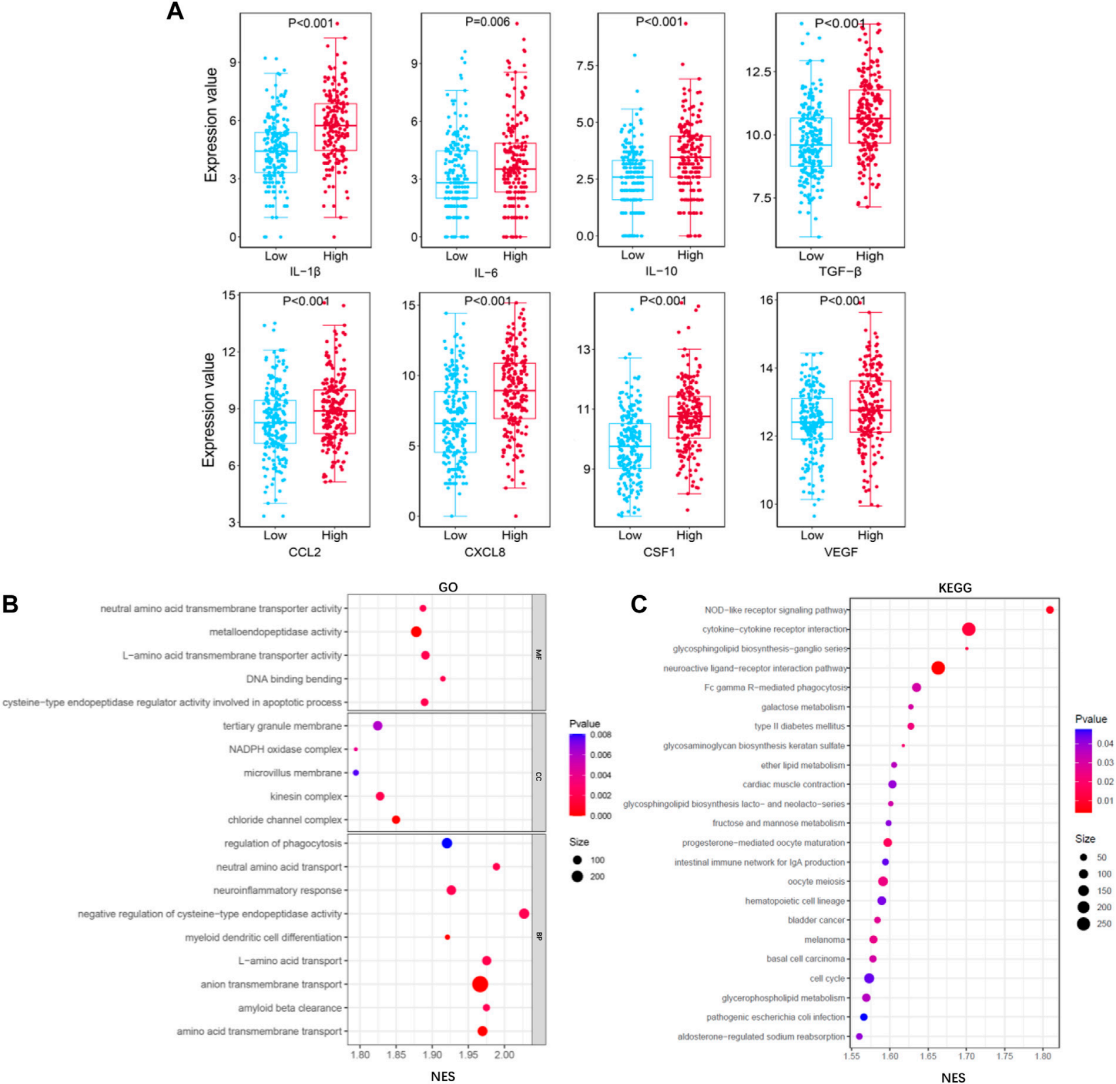
Functional analysis for TIMGs. **(A)** Comparison of the expression value of cytokines (IL-1β, IL-6, IL-10, TGF-β, CCL2, CXCL8, CSF-1, and VEGF) with different groups of TIMSig in the TCGA cohort. **(B)** GOfunction analysis of TIMSig in the TCGA cohort. **(C)** KEGG pathway analysis of TIMSig in the TCGA cohort.

**FIGURE 10:**
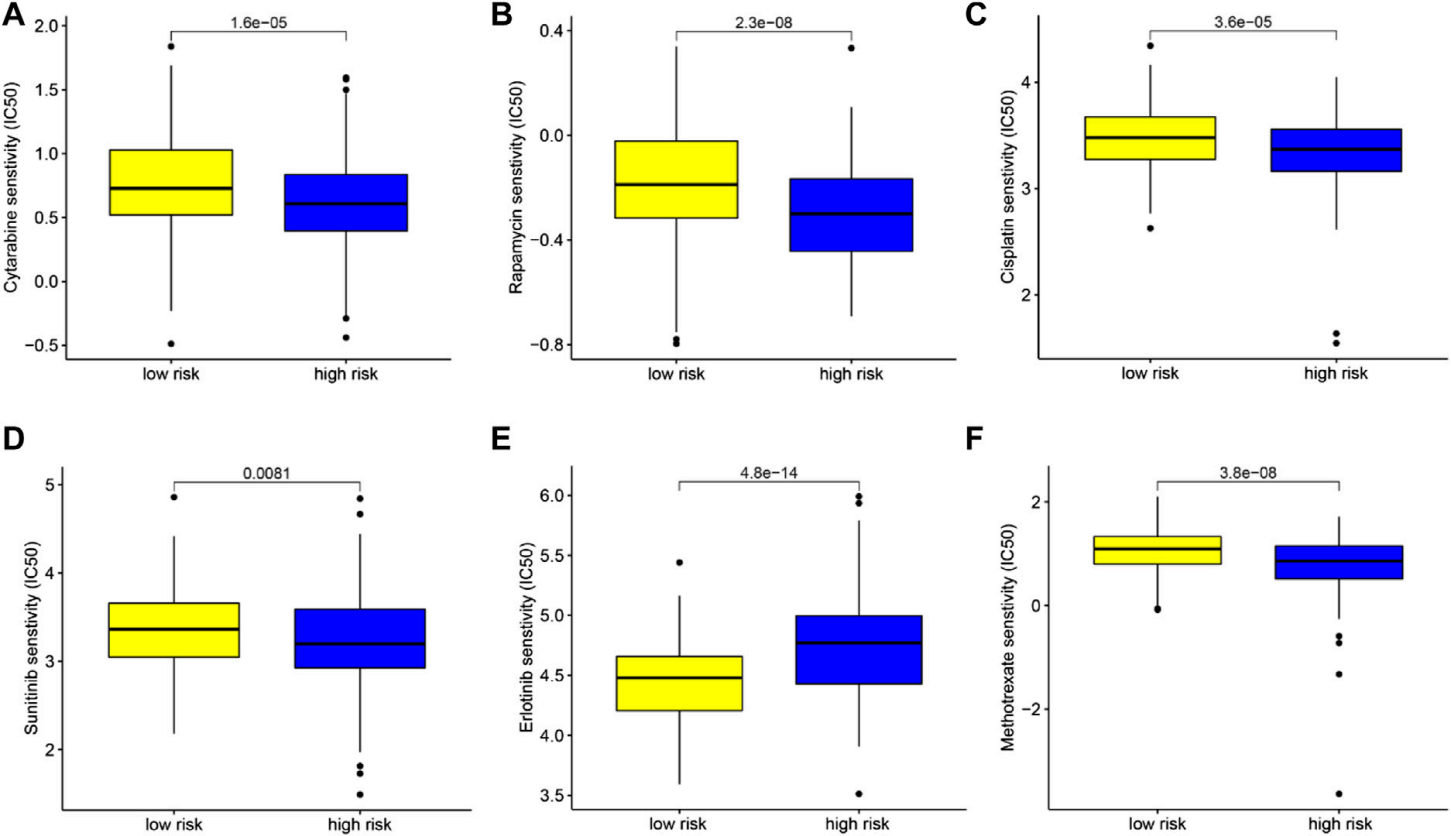
Chemotherapeutic drug sensitivity analysis based on TIMSig. **(A–F)** Comparison of the IC50 levels (cytarabine, rapamycin, cisplatin, sunitinib, erlotinib, and methotrexate) with different groups of TIMSig in the TCGA cohort.

The publisher apologizes for this mistake. The original version of this article has been updated.

## Publisher’s note

All claims expressed in this article are solely those of the authors and do not necessarily represent those of their affiliated organizations, or those of the publisher, the editors, and the reviewers. Any product that may be evaluated in this article, or claim that may be made by its manufacturer, is not guaranteed or endorsed by the publisher.

