# Supplementary material for: Construction of a tumor immune infiltration macrophage signature for predicting prognosis and immunotherapy response in liver cancer
Source: Front Mol Biosci. 2022 Sep 2;9:983840. doi: 10.3389/fmolb.2022.983840 (PMC9479109; doi:10.3389/fmolb.2022.983840)
Supplement: Supplementary file 1 [file DataSheet1.PDF]

Supplementary Material

Supplementary Figure 1 LASSO Cox regression analysis. (A) LASSO coefficient profiles. (B) The optimal penalty parameter lambda of LASSO Cox regression.

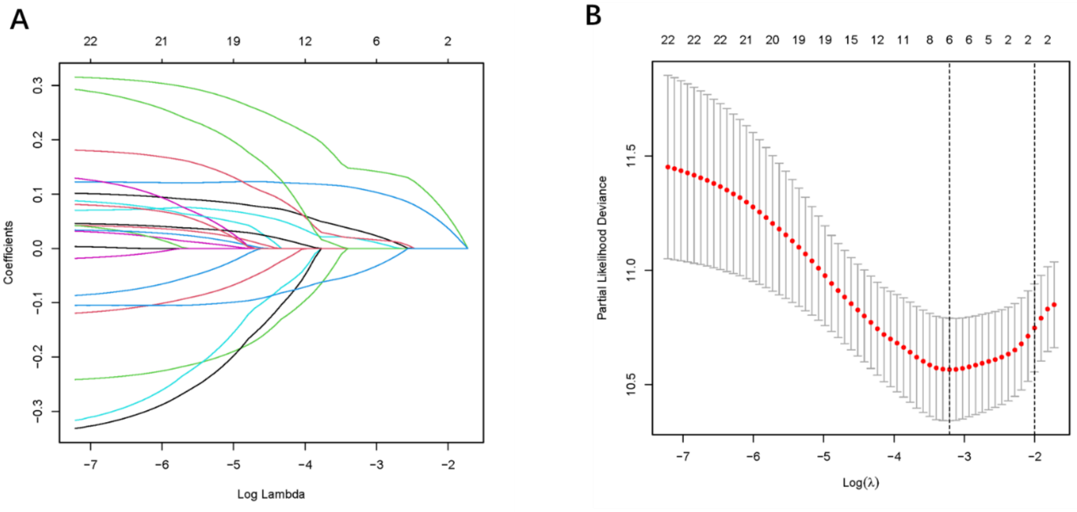

Supplementary Figure 2 (A-C) The immunohistochemistry staining for S100A9, SLC22A15, TRIM54 from HPA database.

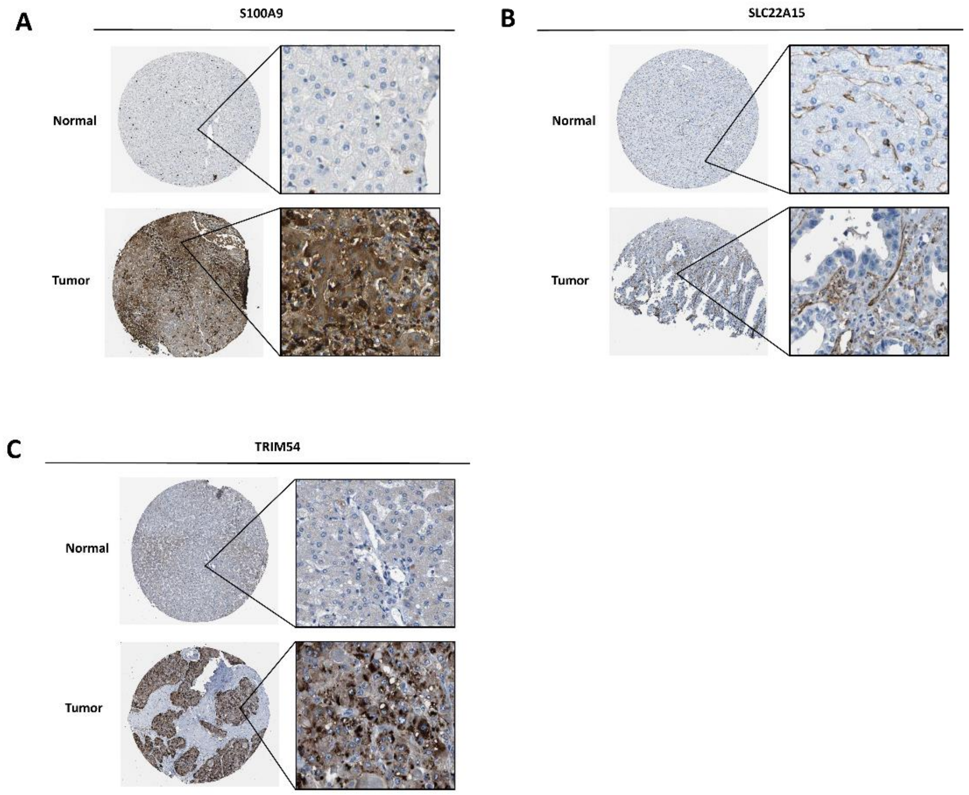

Supplementary Figure 3 (A-D) Differential expression of TIMGs in pan-cancer with TCGA cancer samples.

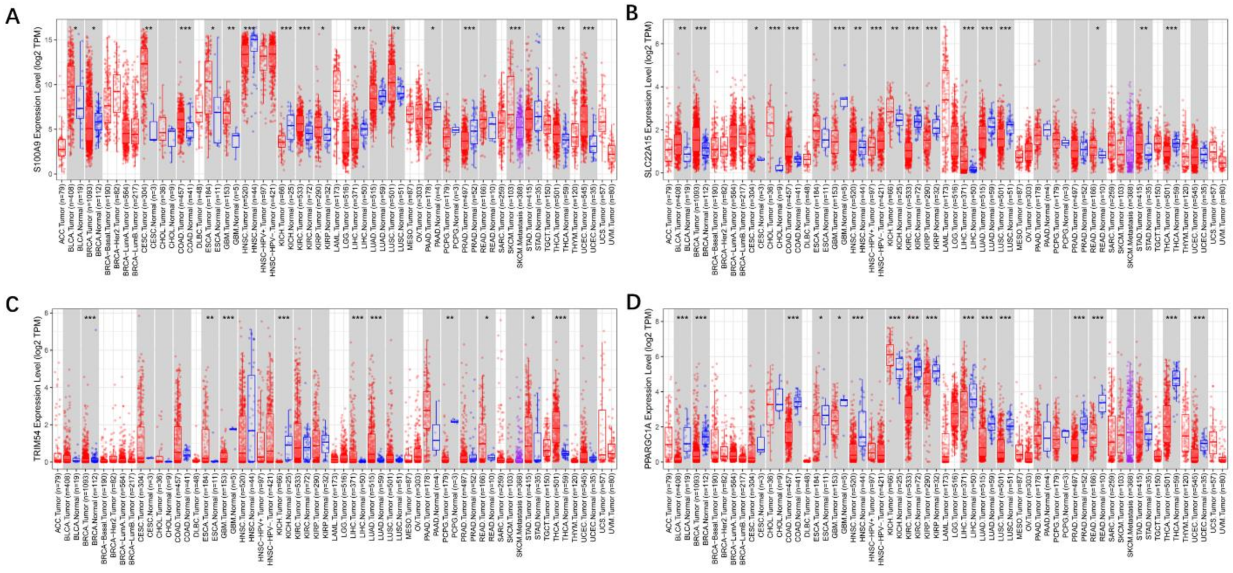

Supplementary Table 1 The Platforms of GEO datasets

No.	Dataset	Platform
1	GSE23371	Affymetrix Human Genome U133 Plus 2.0 Array
2	GSE27291	Affymetrix Human Genome U133 Plus 2.0 Array
3	GSE157737	Affymetrix Human Gene 1.0 ST Array [transcript (gene) version]
4	GSE27838	Affymetrix Human Genome U133 Plus 2.0 Array
5	GSE37750	Affymetrix Human Genome U133 Plus 2.0 Array
6	GSE8059	Affymetrix Human Genome U133 Plus 2.0 Array
7	GSE51540	Affymetrix Human Genome U133 Plus 2.0 Array
8	GSE149425	Affymetrix Mouse Genome 430A 2.0 Array
9	GSE104852	Affymetrix Mouse Gene 1.0 ST Array [transcript (gene) version]
10	GSE52156	Illumina HumanHT-12 V4.0 expression beadchip
11	GSE155148	Affymetrix Mouse Genome 430 2.0 Array
12	GSE83441	Affymetrix Human Genome U133 Plus 2.0 Array
13	GSE42058	Affymetrix Human Genome U133 Plus 2.0 Array
14	GSE106932	Illumina HumanHT-12 V4.0 expression beadchip
15	GSE28726	Affymetrix Human Genome U133 Plus 2.0 Array
16	GSE158792	Phalanx Human OneArray Ver. 7 Release 1
17	GSE56755	Affymetrix Mouse Genome 430 2.0 Array
18	GSE75829	Agilent-014850 Whole Human Genome Microarray 4x44K G4112F (Probe Name version)
19	GSE14520	Affymetrix Human Genome U133A 2.0 Array<br>Affymetrix HT Human Genome U133A Array
20	GSE140901	nCounter PanCancer Immune Profiling Panel

Supplementary Table 2 Basic clinical characteristics in the training and test cohort

Variable		Training cohort (n=202)	Test cohort (n=202)
Stage	I/II	148 (151.5)	155 (151.5)
	II/IV	54 (50.5)	47 (50.5)
Gender	Female	65 (70.0)	75 (70.0)
	Male	137 (132.0)	127 (132.0)
Age	>65	77 (77.5)	78 (77.5)
	≤65	125 (124.5)	124 (124.5)
BMI	>30	43 (39.5)	36 (39.5)
	≤30	159 (162.5)	166 (162.5)
